# N300 and Social Affordances: A Study with a Real Person and a Dummy as Stimuli

**DOI:** 10.1371/journal.pone.0047922

**Published:** 2012-10-31

**Authors:** J. Bruno Debruille, Mathieu B. Brodeur, Carolina Franco Porras

**Affiliations:** 1 Douglas Mental Health University Institute, Montréal, Québec, Canada; 2 Department of Psychiatry, McGill University, Montréal, Québec, Canada; 3 Department of Neurology and Neurosurgery, McGill University, Montréal, Québec, Canada; 4 Centre d’Higiene Mental Les Corts, Barcelona, Spain; University of Bologna, Italy

## Abstract

Pictures of objects have been shown to automatically activate affordances, that is, actions that could be performed with the object. Similarly, pictures of faces are likely to activate social affordances, that is, interactions that would be possible with the person whose face is being presented. Most interestingly, if it is the face of a real person that is shown, one particular type of social interactions can even be *carried out* while event-related potentials (ERPs) are recorded. Indeed, subtle eye movements can be made to achieve an eye contact with the person with minimal artefacts on the EEG. The present study thus used the face of a real person to explore the electrophysiological correlates of affordances in a situation where some of them (i.e., eye contacts) are actually performed. The ERPs this person elicited were compared to those evoked by another 3D stimulus: a real dummy, and thus by a stimulus that should also automatically activate eye contact affordances but with which such affordances could then be inhibited since they cannot be carried out with an object. The photos of the person and of the dummy were used as matching stimuli that should not activate social affordances as strongly as the two 3D stimuli and for which social affordances cannot be carried out. The fronto-central N300s to the real dummy were found of greater amplitudes than those to the photos and to the real person. We propose that these greater N300s index the greater inhibition needed after the stronger activations of affordances induced by this 3D stimulus than by the photos. Such an inhibition would not have occurred in the case of the real person because eye contacts were carried out.

## Introduction

In their recent book chapter entitled “An ecological theory of face perception”, Zebrowitz et al. [1] include social affordances in the processes triggered by the occurrence of a face. They defined this particular type of affordances as the opportunities for acting or being acted upon that are provided by other people, thus including the appeal to make eye contact, smile, say hello, shake hands, start a conversation etc. More generally, affordance is a concept initially developed by Gibson [2], [3] and well illustrated by his quotation from Koffka “Each thing says what it is… a fruit says ‘eat me’; water says ‘drink me’; thunder says ‘fear me’”. In other terms, affordances are the possibilities of actions that objects and people prime. They can be considered as part of the coding of the meaning of an object within theories of embodied or grounded cognition (e.g., [4], [5]). Although originally referring exclusively to properties of objects, the term “affordance” will be used here to designate both these properties and their corresponding neurophysiological counterparts.

Evidence for affordances was found by presenting pictures of objects that could be grasped with the right or the left hand. Pictures of objects with the handle oriented to the right were responded to faster with the right hand, whereas pictures of objects with handle oriented to the left were responded faster with the left hand [6]. Further testing showed that this decrease of reaction time occurs independently from spatial attention [7] and regardless of the instructions ([6]; but see [8]). Affordances are thus activated in an automatic fashion, even when there is no intention to act, a conclusion further supported by the activation of affordances by stimuli that are briefly presented or masked ([9, 6 & 10].

Affordances of objects have been studied within well-established frameworks separating their processing from the one leading to the recognition of the object (e.g., [11]). According to these frameworks, vision-for-perception and vision-for-action are mediated by two distinct streams. Both process the structure of objects and their spatial locations but they produce quite different outputs (see [12] for a review of evidence supporting this model). The dorsal ‘action’ stream’ goes from occipital to posterior parietal areas and performs the visuomotor transformations necessary to act upon the objects and to control actions. It codes information in an egocentric frame of reference [13]. The ventral ‘perceptual’ stream goes from occipital to temporal areas and includes the representations needed for recognition and identification. It codes information in an allocentric frame of reference. Noteworthy, it is the activity of the ventral pathway that has mainly been explored when using faces of people as stimuli. Nevertheless, people do not only trigger perceptions but also actions, such as speech, facial expressions, eye movements and gestures. Face stimuli activate the dorsal pathway, which thus has to be studied.

Numerous brain imaging studies provided support to this idea of a vision-to-perception and vision-to-action segregation. Stimuli with high motor-based properties associated to specific hand movements, such as tools, were found to activate specific brain areas to a greater extent than stimuli with low motor-based properties, such as houses and animals. These brain areas included the left ventral premotor area and the left posterior parietal lobe (e.g., [14 & 15]. Meanwhile, imagining tools lead to more ventral premotor activities than when imagining control stimuli [16, 17 & 18]. Using different experimental designs, affordance-related activities were also described in several foci localized in the inferior parietal cortex, the inferior frontal gyrus, the supplementary motor area, the premotor cortex and the cerebellum [19]. Most of these areas are in the frontoparietal and temporal network that Creem-Regehr and Lee [20] described as being activated by the functional utility of graspable objects.

Electrophysiological studies also brought interesting insights in the mechanisms of affordances to objects, especially in the temporal dynamics of their activations. Kiefer et al. [21] recorded the event-related potentials (ERPs) of subjects while they were sequentially shown two objects, which had to be named. These two objects were associated with congruent or incongruent actions. The ERPs elicited by the second object included differences over two waveforms. The amplitude of the first waveform, the P1 (85–115 ms), was smaller for incongruent than for congruent actions. Its generators were mostly localized in the right inferior parietal, right postcentral and right precentral gyri. The amplitudes of the second waveform, a negative deflection occurring between 380 and 480 ms, termed N400, were larger for incongruent actions, an effect seen by the authors as reflecting the integration of modality-specific conceptual features from the sensory and motor systems. Interestingly, van Elk et al. [22] also reported an N400 (380–450 ms) effect that could be related to affordance. However, it was in the direction opposite that of the N400 effect found by Kiefer et al. [21]. The task used could be responsible for this discrepancy. Van Elke et al.’s [22] subjects were instructed to get prepared for an action that was congruent with the object shown, or incongruent. Preparation of congruent actions elicited stronger anterior N400, possibly originating from the left dorsal premotor area. This effect was attributed by the authors to the retrieval and activation of congruent actions, rather than to a later integration processes.

Thus, the discrepancies existing between the N400 effects of the two studies could be due to the important differences between the experimental designs used. In Kiefer et al.’s [21] study the action required was naming. It thus had no relation with the affordances that were manipulated. In Van Elk et al’s [22] study, the action was related with the affordances activated by the object in the congruent condition. Thus, it seems that this latter study was more appropriate to explore the timing of the activations of affordances. However, the action that had to be performed had to be *withheld* until cues occurred at least 2500 ms after the onset of each object’s picture eliciting the ERPs under study. Otherwise, muscle activity would have artifacted the ERPs. The N400 effect obtained could thus be interpreted as being due to that withholding, the intensity of which could be proportional to the activation of the affordance triggered by the stimulus. To explore the timing of the activation of affordances, it would thus be ideal if subjects could carry out an activated affordance without delay and without creating motor artefacts on ERPs.

The face of a real person could provide these particular conditions to the extent that subjects can make an eye contact with very subtle eye movements. One of the most basic social affordances triggered by persons could thus be carried out with minimal motor artefacts, especially if the eyes of subjects are already looking in the direction of the eyes of the person that will be presented. However, although it is technically possible to illuminate the face of a person suddenly in order to display it as a stimulus, it is very difficult to present 40 different persons to each subject in these conditions. Thus, to obtain the particular conditions described, the presentation of the face of the same person has to be repeated during an entire block. This raises two important problems. The first is that the results that will be obtained may then depend on that person and not be generalizable to other face stimuli. However, this problem may be solved by using, as a control, the photo of this person in the same conditions as this person appears as a stimulus. By using such a photo, the activity due to the particular features of this person will be controlled for. The second issue is that repeating over and over the presentation of the same stimulus eliminates almost certainly the possibility of obtaining a sizable N400 potential. Indeed, in such an experimental design, the re-appearance of the face is completely expected by the subject and accurate predictions are well-known to suppress N400 activity [23]. However, if affordances are automatically activated and if some anterior components of the N400s reflect this activation, as suggested by van Elk et al. [22], it seems that the presentation of a person with whom eye contact can be made should evoke some N400 activity. The first aim of the present experiment was to test this prediction and thus to explore the ERPs elicited by a stimulus for which part of the affordances can be acted out and to see whether it would elicit a larger N400 potential than a 3D dummy and the photos of these two stimuli, with which the same affordances are inappropriate, as no real eye contact can be made with these latter stimuli.

On the other hand, it is of particular interest that stimuli capable of directly evoking affordances, like pictures of objects and faces, elicit a particular brain potential in addition to the N400. This potential is a negative going deflection which peaks around 300 ms after the onset of an image of an object or a face [24, 25, 26 & 27]. This N300 has a fronto-central distribution on the scalp, which could suggest that it is related to plans of actions, such as affordances. Nevertheless, this fronto-central N300 occurs together with a positive going wave maximal at occipito-temporal scalp sites and several works have shown that at least some of the generators of this N300 are located in the occipito-temporal cortex [28, 29, 30, 31 & 32]. The processes performed by these generators would be involved in the selection of the object model that best matches the percept (see for instance [33]). In other words, the N300 would index processes involved in the recognition of visual objects. Thus, at first sight, it appears unlikely that N300 processes could be involved in the coding of affordances. However, this conclusion could be premature for two reasons. First, the existence of occipito-temporal generators does not exclude the possibility of concurrent prefrontal generators. Second, even if the N300 only had occipito-temporal generators, some of these generators could be involved in the coding of actions, as suggested by the works of Kable and Chatterjee [34] and Wiggett and Downing [35]). A second aim of the present exploratory study was thus to see whether the N300 would be modulated by acted affordances.

A third goal was to see whether the early differences found by Kiefer et al. [21] in the time window of the P1 component, could be replicated using faces as stimuli.

**Figure 1:**
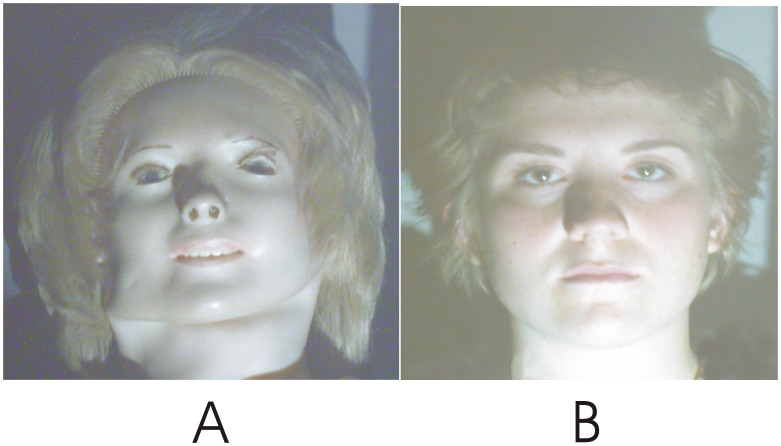
Photo of the face of the person and of the face of the dummy used as stimuli in the two live condition’s blocks. These photos were taken from the perspective of the subjects and were also used as stimuli in the photo condition’s blocks.

To achieve our three goals, we used, as mentioned, the face of a real person with whom a particular social affordance could be carried out during its presentation through eye contacts. The first control stimulus was the face of a real dummy, which clearly appeared as such, and with which social contact was impossible. It was used as a stimulus the visual appearance of which roughly matched that of the real person (see Fig. 1). The photos of these two stimuli were used as other control stimuli and as 2D stimuli which should a priori activate affordances a) in a weaker way than their 3D counterpart and b) that should not be acted upon as the system is likely to identify that they are not a real person with which social interactions would be possible.

## Methods

### 1. Participants

20 (9 men) right-handed individuals with a mean age of 25 years (range 18 to 35) participated in the study. One participant had completed high school, eight had a college level of education and eleven had some university education. Participants did not report any neurological or psychiatric problem and signed an informed consent form approved by the Douglas Institute Research and Ethics Board. This Board, which follows the principles expressed in the declaration of Helsinki, also approved the study itself.

### 2. Stimuli

A dummy employed for the training of medical students was used. Figure 1a shows the photo of this dummy taken from the point of view of the participants in the live condition. This dummy was chosen so that its resemblance with a real person was limited. This was done in order to have a sufficient matching with the physical features of the real face but to prevent the possibility of mistaking the dummy for a real person. The dummy was thus chosen to be well-recognized as such. The photo of the face of the real person taken in the same condition is shown in Figure 1b. This person was asked to maintain a facial expression the valence of which was as close as possible to that of the dummy in order to prevent ERP activities to differ because of valence. Noticeably, eye contacts with the person stimulus occurred during the live condition. This was confirmed by both the confederate whose face was used as a stimulus, and by each participant. It has to be noted that it is not yet possible to check the occurrence of eye contacts by measuring the eye movements of the participant. Small changes of the position of the head and of the eyes of the confederate naturally occurred during the experiment. These moves of the “target” make the measurement of the eye movements of the participant irrelevant. Both faces were lit with a slide projector which included no slide but whose lens was equipped with a Displaytech ferroelectric liquid-crystal shutter driven by the TTL signals of our stimulus presentation computer. This setting allowed a sudden (i. e., within 50 microseconds) appearance of the entire face, which remained lit for only 200 ms, in order to prevent bigger eye movements (that would explore parts of the face other than the eye region). All faces were at a 1.5 m distance from the eyes of the participant, sustaining an 8 degrees vertical and a 6 degrees horizontal visual angle. The photos of the two stimuli (i. e., Fig. 1a & 1b) were presented on a computer screen. Their sizes were adjusted to match that of the 3D faces. Their contrast and color were adjusted by manipulating the monitor display till they appeared identical to that of the 3D stimuli in the live condition. We used a Lunasix 3 photometer to adjust the luminance of the screen so that the face pictures provided the same amount of light as the 3D faces in live condition. Timing of these computer displays were adjusted to match those of the live presentation of the real faces, except for the onset and offset of the photos, which followed the refresh rate of the computer screen display, from top to bottom, which took 13 milliseconds.

### 3. Procedure

The stimuli were shown in 4 blocks which order was counterbalanced across participants. In each block, one of the 4 stimuli, the real dummy, the real face, the dummy’s face photo, or the photo of the real face, was presented 60 times. Intertrial intervals varied randomly between 1.5 and 2 seconds to prevent the development of contingent negative variations. For the real dummy and the real person blocks, the participants were seated comfortably in a dimly lit room in front of a one-way mirror. The dummy/person was placed on the other side of the mirror. Participants could only see the dummy/person when the liquid crystals of the shutter placed inside the slide projector (see above) were transparent. In the photo conditions, participants saw the photo of the two stimuli taken in live conditions on a computer screen. In all blocks, the task was to pay attention to the displays and to keep the gaze at the eye area of the face stimuli so as to minimize the eye movements necessary to make the eye contact with the real person. Given the use of block presentation, participants knew what the next 59 occurrences were going to be. It was easy for them to plan eye contacts with the real person and thus to develop the strategy on focus here.

### 4. Data Acquisition

The electroencephalogram (EEG) was recorded with tin electrodes mounted in an elastic cap (Electrocap International) at 28 of the sites of the extended International 10–20 System [36]. The reference electrode was placed on the left ear lobe. The active electrode sites were grouped in a sagittal subset, which included Fz, FCz, Cz, and Pz; a parasagittal subset, comprising FP1/2, F3/4, FC3/4, C3/4, CP3/4, P3/4, and O1/2; and a lateral subset encompassing F7/8, FT7/8, T3/4, TP7/8, and T5/6. In addition, two active electrodes were placed below each eye in order to allow the monitoring of vertical eye movements. Horizontal eyes movements were monitored by comparing signals from F7 and F8. The EEG was amplified 20.000 times by Contact Precision amplifiers. High and low-pass filter half-amplitude cut-offs were set at.01 and 100 Hz, respectively, with an additional electronic notch filter to remove 60 Hz contamination. Signals were then digitized on-line at a sampling rate of 256 Hz and stored for subsequent averaging using the Instep (version 4.3) software package.

### 5. Data Processing

EEG epochs of the last 50 trials of each block were examined in order to focus only on trials for which habituation was maximal. Trials contaminated by eye movements, excessive myogram, amplifier saturations or analog to digital clipping were removed offline by setting automatic rejection criteria. Trials for which analog to digital clipping exceeded a 100 ms duration and electrodes for which amplitude exceeded ±100 µV were discarded. On average, this led to the exclusion of 12% of the trials. ERPs to each of the four face stimuli were computed by averaging the 1000 ms EEG epochs of these trials in each experimental condition (i. e., of each block), using a –200 to 0 ms baseline before stimulus onset.

### 6. Measures and Analyses

**Figure 2:**
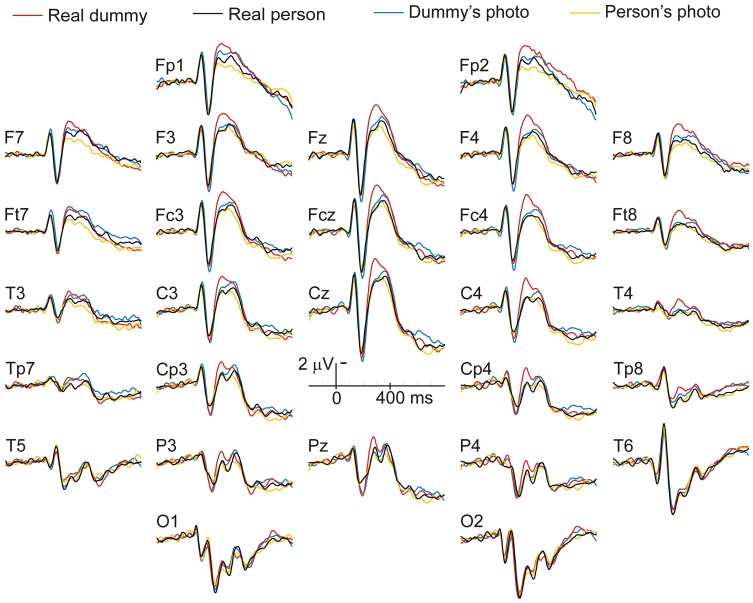
Grand average (n = 20) of the event-related brain potentials elicited in each of the four blocks: that of the real person, that of the real dummy, that of the person’s photo and that of the dummy’s photo.

The comparison of the ERPs to the real dummy with those to the real face (Fig. 2) led to sizeable differences in the time window of the fronto-central N300. They started around 270 ms post onset, were maximal at 300 ms and disappeared around 400 ms. The mean voltage of the ERPs was measured in the 270–400 ms time-window, respective to the baseline. ERP differences were detected by visual inspection in two other time-windows. The first consisted of more negative ERPs for the dummy and its photo than for the face and its photo between 125 and 170 ms particularly at P4 and CP4. In the second, ERPs were more positive for the dummy and its photo than for the face and its photo between 170 and 230 ms, especially at Pz. Mean voltages of ERPs within these two time-windows were measured and analyzed to generate a priori hypotheses for future studies.

The mean voltage amplitudes of the ERPs in the 125–170, 170–230 and 270–400 ms time windows were each entered in three repeated-measures ANOVAs using a multivariate approach. For the ANOVAs dedicated to the sagittal subset of electrodes, three within-subjects factors were used, presentation type (real vs. photo), stimulus type (person vs. dummy) and electrode site. For the ANOVAs dedicated to the parasagittal and the lateral subset of electrodes, a fourth factor was added, the hemiscalp (right vs. left). The Greenhouse and Geisser [37] correction for lack of sphericity was used to correct degrees of freedom for the factor that had more than two levels (i.e., electrode site). In this case, original degrees of freedom are reported with corrected *p* values.

## Results

The inspection of the early ERPs at occipito-temporal electrode sites revealed no sizeable differences between conditions (see Fig. 2), neither for the positive deflections peaking a little before 100 ms, the P1s, for the negative deflections that follows, the N170s, or in the time region of the early posterior negativities, the EPNs. The inspection at anterior scalp sites also failed to detect any differences in the time windows of the N1 and P2 deflections.

ANOVAs conducted on the mean voltages measured in the 270–400 ms time-windows revealed a significant effect of stimulus type at the sagittal, *F*(1,19) = 6.65, *MSE* = 66.50, *p* = .018, parasagittal, *F*(1,19) = 8.87, *MSE* = 156.44, *p* = .008, and lateral, *F*(1,19) = 8.04, *MSE* = 76.05, *p* = .011, subsets. When focusing on the real stimuli to test the main prediction, the dummy was found to elicit larger N300s than the person at the sagittal, *F*(1,19) = 6.8, *p* = .017, parasagittal, *F*(1,19) = 9.24, *p* = .007, and lateral, *F*(1,19) = 8.55, *p* = .009, subsets. There was no significant interaction between stimulus type and electrode site or hemiscalp, only trends (*p* = .1). When focusing on the photos to test the absence of an effect on the N300s, no effect of stimulus type was found at the sagittal, *F*(1,19) = .67, *p* = .4, parasagittal *F*(1,19) = .97, *p* = .34, and lateral subset *F*(1,19) = 1.3, *p* = .27. Stimulus type did not interact with electrode or hemiscalp. On the other hand, N300s elicited by the real dummy appeared somewhat larger than the N300s elicited by the photo of the dummy (Fig. 2). These differences were analyzed to test whether 3D stimuli with which no affordance can be carried out elicit larger N300s than 2D stimuli. The differences were not significant on the sagittal *F*(1,19) = 1.4, *p* = .26, parasagittal *F*(1,19) = .96, *p* = .34 and lateral subset *F*(1,19) = .52, *p* = .48. Presentation type did not interact with electrode or hemiscalp.

The ANOVAs run with the mean voltages of the ERPs in the 125–170 ms time windows revealed a significant interaction between stimulus type (dummy vs. person) and electrode at the sagittal subset of electrodes, *F*(3,57) = 9.05, *MSE* = 10.97, *p* = .002. The posthoc test conducted at Pz to look for the source of the interaction revealed a significant effect of stimulus type, *F*(1,19) = 5.04, *MSE* = 48.87, *p* = .037, suggesting an effect in the downhill slope to the P2. The ANOVAs made for the parasagittal subset revealed an interaction of stimulus type with electrode and hemiscalp, *F*(6,114) = 4.26, *MSE* = 3.65. *p* = .013. Posthoc run at P4 to identify the source of this interaction revealed that the ERPs to the dummy were more negative than those to the person, *F*(1,19) = 10.06, *MSE* = 92.66, *p* = .005. The ANOVA made for the lateral subset of electrodes did not reveal any effect or tendency.

In the 170–230 ms time window, the ANOVA for the sagittal subset detected a just significant effect of stimulus type, *F*(1,19) = 4.55, *MSE* = 37.54, *p* = .048. To generate a priori hypotheses for future studies, it was tested at Pz, *F*(1,19) = 4.95, *MSE* = 17.53, *p* = .025, where it appeared to be larger. The ANOVAs conducted for the parasagittal and for the lateral subsets failed to detect any significant effect.

## Discussion

In this study, four different stimuli were used, a real unknown woman, the dummy of an unknown woman, and the photo of these two stimuli. Social affordances were acted out through eye contact with the real person. Each of these four stimuli was presented repeatedly in one block while subjects had to pay attention. All stimuli generated a large negative deflection maximal at 300 ms post onset over fronto-central scalp sites. None of these stimuli generated a sizable N400 potential as no peak was observed on the downhill slope from the N300s to the P600s and as the ERPs elicited by the four stimuli did not differ in the N400 time window. Meanwhile, in the time-window of the N300s, the ERPs elicited by the real dummy were significantly more negative than those evoked by the real person and the photos. Finally, ERPs in early time windows (125–170 and 170–230 ms) were found to depend on the nature of the stimulus (dummy or person) and not on the presentation type (real or photos). Although these early differences were maximal at parietal sites, as in Kiefer et al. (2011), they were not as early as the effects found by this team, which were in a 85–115 ms time window.

The absence of an N400 peak and of an N400 effect is not surprising. It is likely to be due to the fact that only one stimulus was presented in each block. The occurrence of each stimulus was thus fully predictable. Anticipation was maximal for all stimulus conditions. Nevertheless, if our four stimuli actually activated affordances to different extents, this N400 absence goes against van Elk et al.’s [22] suggestion that the amplitude of the anterior part of this potential is proportional to the activation of affordance.

As to the fronto-central N300s obtained here, several possibilities have first to be eliminated. These N300s could not be late mismatch N200s (see for instance [38]) since, each stimulus was fully predictable and identical to the preceding one as it was the only stimulus presented in its block. Therefore, its occurrence always matched the expectation of the subjects and the previous stimulation. Second, the N300 effect, that is, the larger N300s observed for the 3D dummy than for the other stimuli, is unlikely to be due to eye movements. This possibility could be evoked due to the presence of this effect at the electrode sites that were the closest to the eyes, Fp1/2. But this possibility can be discarded because the size of the effect does not decrease rapidly along the antero-posterior axis. Its amplitude at central site (Cz) is similar to that observed at Fp1/2.

Third, the N300 effect is probably not a type of N400 effect whose peak latency would have been shortened by the intense repetition of the stimuli, as it was the case in [39, 40 & 41]. Indeed, the N300 effect of the present study appears to have a fronto-central maximum whereas the early N400 effects obtained in intense repetition conditions had a classical centro-parietal maximum. Another fact argues against seeing the present N300 effect as an early N400 effect. To the extent that it was obtained between a pseudo person and a person, it could have been compared to the effect obtained between pseudo words and words at high repetition rates. However, this latter lexicality effect was maximum around 400 ms, despite massive repetitions [42], thus about 100 ms later than the present N300 effect. Fourth, the possibility that the N300 effect could be due to a larger effect of repetition for the three stimuli than for the 3D dummy can also be discarded. The repetition of the presentations of a stimulus is known to induce a decrease of N400s and N400-like components it elicits. However, this decrease is large only between the first and the second presentation. There is no important decrease at further presentations [43, 44, 45, 46 & 47]. Here, the first ten presentations were not used to compute the ERPs.

Fifth, the N300 effect observed here is also unlikely to be related to an attention N2 (the N2b, [48]) as it seems that a 3D dummy cannot trigger more attention than a real person. However, it could be argued that the dummy face triggered more visual attention because it is novel, looks kind of scary (Fig. 1) and that, in daily life, we do not have much experience with observing this type of object. Nevertheless, if subjects had been more attentive during the block of the 3D dummy, larger P1s, N1s and LPCs should have been observed. This was not the case. It appears unlikely that a greater attention to the 3D dummy would have resulted in the exclusive enhancement of the fronto-central N300s. Moreover, although it may be possible that the 3D dummy triggered more attention than the other stimuli at its first 10 presentations, these presentations were not used to compute ERPs. Finally, if anterior N300s were sensitive to attention and if attention actually differed across stimuli, N300s should have been larger for the real person than for its photo; this was not the case.

Given that the dummy may look kind of scary, one could also wonder whether the N300 effect could be related to a perception of its negative emotional valence. However, this idea can also be discarded since if it were true, then the N300s to the 2D dummy would also have been greater than those to the 2D person’s face. Nevertheless, it could be argued that 3Ds might contribute to make the real dummy scarier than its photo. However, even this possibility could not account for the larger fronto-central N300s it elicited. On the contrary, negative facial expressions induce more positive anterior ERPs in these time windows than positive expressions (for a review, see [49]).

Another account of the greater N300s generated by the 3D dummy could have been based on the idea that the N300 reflects the activation of image specific semantic properties as suggested by [26 & 50]. Indeed, it could be argued that the dummy activate more semantic features as it represents a person *and* is some kind of art work, which, as such, conveys additional semantics. However, this account is undermined by the absence of significant N300 differences between the photo of the dummy and the photo of the person. It seems that a difference similar to that observed between the real dummy and the real person should have been observed between their photos, according to that activation account. This was not the case. This absence of difference between the two photos also undermines an account of the present N300 difference in terms of processes that would match the visual percept with object (i.e., face) representations as suggested by [33].

Finally, before discussing the possibility of a relation between the N300 effect observed here and social affordances, one can wonder whether the large fronto-central N300s elicited by the 3D dummy could index a greater activation of other types of affordances. These activations would be greater for this stimulus since it is the only one that can be grasped. Indeed, we do not usually grasp the face of other people. It is thus unlikely that the face of the real person activated these affordances. This explanation would also account for the small fronto-central N300s of the photos, which are likely to activate action affordances to a smaller extent than 3D stimuli. However, as mentioned in the introduction, 2D pictures of objects have been shown to activate action affordances. Therefore, according to this action affordance account, greater N300 should have been found for the 2D dummy than to the 2D real face. This was not the case. Moreover, in most action affordance studies, the stimuli are objects that are usually grasped in every-day life (e.g., a cup with a handle, a screw-driver). Grasping the face of a dummy may not be the straightforward type of affordances triggered by this “object”, which is essentially built to *look* like a face and thus built to be looked at, and not to be grasped, poked or thrown… Faces are, first and foremost, social media. Therefore, it appears very unlikely that the large fronto-central N300s elicited by the dummy index the activation of action affordances such as graspability.

Therefore, the fact that these effects were observed between stimuli differing in their *social* affordances can be used to support the idea that some components of fronto-central N300 potentials indexes these affordances. However, the effects obtained were in a surprising direction. The N300s elicited by the real face, and thus by the stimuli the most capable of activating social affordances, were not the largest. They were in fact significantly smaller than those elicited by the real dummy, which were greater than those elicited by the three other stimuli. An account for this unexpected direction of effects can be based on two plausible ideas. First, 3D stimuli, namely the real face and the real dummy, induce greater activation of affordances than the photos. Second, the automatic activation of affordance by all these face stimuli is inappropriate, except in the case of the real face, with which social affordances could be and were carried out through eye contacts. Thus, there may be some inhibition of these affordances going on after their early automatic activation. Given that these inhibitions should be proportional to the activations, they should be greater for the 3D dummy than for the photos. Accordingly, the greater N300s elicited by this latter stimulus could index the inhibition of the affordances it strongly activated. The smaller N300s elicited by the real face would be due to the lack of the need for inhibition, since affordances could be acted out in the case of this stimulus. The small N300s elicited by the photos would index the weak inhibition required when affordances are not as strongly activated as they would be by a 3D stimulus.

This inhibition interpretation contrasts with the idea that larger late negativities index the activation of more affordances. However, it may not be in contradiction with the results of previous ERP studies, such as with the larger N400s found by Kiefer et al. [21]. These larger N400s were elicited by pictures of objects associated to actions that were incongruent with the actions associated to the preceding stimulus. These additional late negative activities could be related to more inhibition of actions representations or affordances. This inhibition would be performed because subjects did not have to perform any of the actions that could be done with the objects used as stimuli. They just had to name the object. So the new affordances that were automatically activated by objects that were incongruent with the preceding ones might have been inhibited.

This inhibition view may allow a reconciliation of these results with the larger N400s found by van Elk et al. [22]. These larger N400s were observed for objects associated to actions that were congruent with the action prepared by subjects before the occurrence of these objects. In this latter study also subjects did not have to act when the object was presented (but later). Thus, the affordance automatically activated by the object might also have to be (temporarily) inhibited to delay immediate action. This inhibition could be greater in the case of congruency because of the fit between the preparation and the stimulus, which should be associated to a greater early automatic activation of affordances. The results of experiments, such as the present one, which includes a condition (e.g., that of the real person), where subjects can immediately carry out the automatically activated affordance may thus then shed a new light on the functional significance of late negativities related to actions.

As mentioned, the inhibition view developed here is based on the assumption that the 3D stimuli activated affordances more strongly than their photos, as it seems plausible that real stimuli activate affordances to a higher level than their pictures do. However, the absence of an ERP effect occurring before the N300 that would substantiate this idea is puzzling. Nevertheless, this would not be the first time ERPs would be blind to automatic activations. For instance, in a recent study [51], no N400 effect was observed in a condition for which subjects successfully make a semantic decision for word stimuli. As a matter of fact, it can be shown that ERPs could be blind to the automatic activations of semantic representations corresponding to meaningful stimuli and that the N400 could be generated by inhibition processes occurring after these activations [25, 52, 53 & 54].

The account developed here may also appear surprising to the extent that it stipulates that the activation of affordances starts before the N300s and that some components of these N300s would index inhibition. Nevertheless, it has to be pointed out that this is exactly what is stipulated for another fronto-central and negative potential: the Go/NoGo N2 (e.g., [55 & 56]). This potential, whose scalp distribution and peak latency are amazingly similar to those of the present N300, has been studied in conditions where participants learned to associate an action to a stimulus and thus in conditions in which the presentation of this stimulus might automatically activate the action (e.g., a button press). The Go/NoGo N2 has been consistently found to be larger in the NoGo conditions and thus when subjects have to prevent this immediate action. The possibility that this Go/NoGo N2 indexes the inhibition of the action plan has thus been extensively discussed (e.g., [57, 58 & 59]). Accordingly, the action plan would be automatically activated by the stimulus and would then be inhibited. A medial frontal cortex structure close to the anterior cingulate cortex could be responsible [60]. This inhibition hypothesis is consistent with one of the functions attributed to this cortex, which is frequently impaired in patients with frontal lesions. These patients precisely tend to “act their affordances” without restriction in what is termed the “utilization behavior”. They have difficulty resisting their impulse to operate or manipulate objects which are in their visual field within reach [61].

An alternative possibility also has to be discussed, the fronto-central difference between the real dummy and the real person could have been due to the attribution of motor properties to the real person, which can move by itself, whereas the dummy can not. However, the direction of these N300 differences pleads against such an interpretation. Fronto-central N300 were larger for the stimulus that can*not* move by itself, and thus for the stimulus activating less of these attributions, whereas it seems that the contrary should have been observed. Again, one would have to use the inhibition idea and propose that the larger N300s to the dummy could index the inhibition of an attribution of motoric properties, which would be automatic and strong for a 3D face-looking stimulus. The small N300s to face photos would again be due to the smaller activation (of such properties) by 2D than by 3D stimuli. Less inhibition would then be required for these latter stimuli. Future studies could thus be devoted to identify ERP components indexing such motoric properties. However, to the extent that faces are social media, it is tempting to think that, in the case of the present experiment, these motoric properties are related to the social interactions that can be initiated by others. Thus, even in this perspective, the larger fronto-central N300s elicited by the dummy could thus still index the inhibition of social interactions. Only, it could be those that could have been acted by the special stimuli used. Interestingly, the study of the mirror neuron system [62] suggests that the two possibilities, that is, coding a (social) action of the self and coding the same action performed by another person, may be similar.

It has to be noted that the idea that the fronto-central N300 indexes the inhibition of affordances only pertains to one of the components of this potential. As such, it would not go against the possibility that other components index other processes, such as a mismatch, as suggested by the results of [24]. In their identity matching experiment, the authors of this study presented faces of known and unknown persons as primes and as target stimuli. In some trials, the target was a different photo of the person presented as a prime and required the ‘match decision’ from participants. In other trials, the two photos were those of different persons, thus requiring the mismatch decision from participants. In both the known and the unknown person conditions, the negative deflections peaking around 300 ms were larger in the case of mismatch. Similar results can be observed in [63 & 64].

Lastly, two incidental findings that have no relation with the functional significance of the N300 but that may be of interest for other research fields may be noted. The N170, a brain potential whose amplitude and occipito-temporal distribution has been found to be specific to faces (see for instance [65 & 66]), was similar for the four stimuli. These results are consistent with those of the first study in which the ERPs elicited by the face of a real person and those evoked by the face of a real dummy were recorded [67]. In contrast, the systematic analyses of the differences detected by visual inspection led to find two parietal differences between the ERPs to the dummy (real and photo) and the ERPs to the person (real and photo): the one already mentioned between 125 and 170 ms and another, occurring between 170 and 230 ms. As these differences were in unexpected time-windows, they could be type I errors. They are mainly reported here to generate a priori hypotheses for future studies. These studies could test, for instance, whether the greater N1 (125–170 ms) and the greater P2 (170–230 ms) for the dummy (real and photo) than for the person’s face (real and photo) depend on the activation of an additional type of representations. Whereas both the dummy and the person’s face would activate representations corresponding to an unknown woman, the dummy would activate, in addition, representations corresponding to objects.

In any case, it may be concluded that the late ERP results of the present study suggest that one of the components of the fronto-central N300 could index the inhibition of social affordances (making eye contact) which have been automatically, but inappropriately, activated before the N300. Brain imaging experiments where affordances can be immediately carried out at stimulus presentation may be necessary to fully study the processing of affordances.
